# Psychological intervention reduces self-reported performance anxiety in high school music students

**DOI:** 10.3389/fpsyg.2015.00195

**Published:** 2015-03-03

**Authors:** Alice M. Braden, Margaret S. Osborne, Sarah J. Wilson

**Affiliations:** ^1^Melbourne School of Psychological Sciences, The University of MelbourneMelbourne, VIC, Australia; ^2^Melbourne Conservatorium of Music, The University of MelbourneMelbourne, VIC, Australia

**Keywords:** performance anxiety, music, adolescents, psychological intervention, performance psychology

## Abstract

Music performance anxiety (MPA) can be distressing for many young people studying music, and may negatively impact upon their ability to cope with the demands and stressors of music education. It can also lead young people to give up music or to develop unhealthy coping habits in their adult music careers. Minimal research has examined the effectiveness of psychological programs to address MPA in young musicians. Sixty-two adolescents were pseudo-randomized to a cognitive behavioral (CB) group-delivered intervention or a waitlist condition. The intervention consisted of psychoeducation, cognitive restructuring and relaxation techniques, identification of strengths, goal-setting, imagery and visualization techniques to support three solo performances in front of judges. Significant reductions in self-rated MPA were found in both groups following the intervention and compared to their baseline MPA. This reduction was maintained at 2-months follow-up. There appeared to be inconsistent effects of the intervention upon judge-rated MPA, however the presence of floor effects precluded meaningful reductions in MPA. There appeared to be no effect of the intervention upon judge-rated performance quality. This study highlights the potential for group-based CB programs to be delivered within school music curricula to help young musicians develop skills to overcome the often debilitating effects of MPA.

## Introduction

Music performance anxiety (MPA) is “the experience of intense and persistent anxious apprehension related to musical performance” (Kenny, 2010, p. 433). It can have a serious negative impact on musicians at any age, with research showing it is present in adult (Van Kemenade et al., 1995; James, 1998), adolescent and child musicians alike (Ryan, 2005; Fehm and Schmidt, 2006; Osborne and Kenny, 2008; Boucher and Ryan, 2011). MPA often leads to poor performance and educational outcomes in music (Kubzansky and Stewart, 1999) and to a lack of enjoyment of the performing experience. This, in turn, can lead to unhealthy coping strategies such as an over-reliance on illicit and licit drugs (West, 2004; Park, 2010) or alcohol (Dobson, 2011), which can compromise or prematurely end promising musical careers (McGinnis and Milling, 2005; Ryan and Andrews, 2009).

Data from child and adolescent musicians has revealed that their experience of MPA is similar in quality and intensity to that experienced by adult musicians (Smith and Rickard, 2004; Osborne et al., 2005; Ryan, 2005; Kenny and Osborne, 2006; Osborne and Kenny, 2008). Given that most professional musicians begin their training before the age of 12 years (Nagel, 1987), there have been calls to include early interventions for MPA within school music curricula, alongside music theory and instrumental technique (Nagel, 2009; Khalsa et al., 2013; Thomas and Nettelbeck, 2014). This notion of early intervention is supported by research revealing that performance anxiety appears to be a topic of concern for young musicians, but one that they feel is not adequately addressed by teachers and institutions (Fehm and Schmidt, 2006; Ryan and Andrews, 2009).

This inadequacy may be partly attributable to the limited number of studies examining the effectiveness of treatment for MPA in musicians, particularly efficacy studies for interventions targeted at younger musicians. There have been a number of recent reviews conducted on existing treatments for MPA, which indicate that a large range of treatment modalities have been developed, namely behavioral, cognitive, cognitive-behavioral, pharmacological, and alternative therapies (Kenny, 2005, 2011; McGinnis and Milling, 2005; Brugues, 2011). Unfortunately, the overall finding from these reviews is that too few studies meet rigorous methodological criteria, limiting the confidence with which these treatments can be recommended to musicians with MPA. For example, many studies have small sample sizes, lack a randomly assigned control group, do not specify the details of treatment, and/or fail to collect follow-up data.

The most promising intervention paradigms that emerge from these meta-reviews involve a combination of cognitive and behavioral techniques, including cognitive restructuring, relaxation, and mental skills training (Harris, 1987; Clark and Agras, 1991; Roland, 1993). This is consistent with Kenny and Osborne's (2006) finding that negative beliefs (e.g., “If I make the slightest mistake, they'll think I'm incompetent and I'll get thrown out of school”) greatly increased the prediction of MPA over trait anxiety and gender alone in high school musicians, highlighting the need to address these beliefs in the treatment of MPA in young musicians. Research with adult musicians has also demonstrated that cognitive restructuring techniques that identify and challenge self-defeating, task-irrelevant thought patterns, and replace them with more adaptive and realistic views, significantly reduce levels of MPA (Sweeney and Horan, 1982; Harris, 1987; Clark and Agras, 1991; Connolly and Williamon, 2004; Buswell, 2006). Osborne et al. (2007) piloted a cognitive behavioral (CB) program incorporating performance psychology techniques for MPA in elite secondary music students and found that all students reported reductions in MPA, however only significant reductions were reported for students who adhered to the program requirements. Su et al. (2010) found that training in a relaxation breathing technique was associated with a decrease in MPA in young performers of different backgrounds.

While such studies have demonstrated the efficacy of CB interventions in reducing self-perceived MPA, there is less evidence that these interventions increase the quality of a musician's performance. It was originally believed that a simple relationship existed between anxiety and performance, namely that anxiety impairs the quality of music performance, summarized by the “inverted U” law of arousal and performance (Samuels and Samuels, 1987; Hancock and Ganey, 2003). It is now understood however, that a moderate amount of anxiety enhances performance when an individual's skill level matches the performance demands of the situation (Jackson and Csikszentmihalyi, 1999) and the individual interprets that anxiety positively (Jones et al., 1993). For example, Hancock and Warm's (1989) dynamic model of stress and sustained attention provides an “extended-U” conceptualization through the inclusion of both physiological *and* psychological adaptability to account for the complexity of stress on human performance capability (Hancock and Ganey, 2003).

Indeed, intervention studies attest to this more complex, extended relationship. Some early studies found that musicians' MPA decreased and performance quality improved following an intervention with psychotherapy (Appel, 1976; Sweeney and Horan, 1982). Similarly, Roland (1993) demonstrated significant reductions in self-reported state anxiety as well as moderate to strong effect sizes for improved performance quality following a CB intervention for MPA in tertiary students. In contrast, other studies have found no improvements in performance quality following behavioral interventions (Wardle, 1975; Mansberger, 1988; Deen, 2000; Reitman, 2001). For example, whilst Osborne et al. (2007) found significant improvement in self-reported MPA following a CB intervention, there appeared to be no effect on performance quality as rated by external judges. Consistent with the dynamic model of stress and performance (Hancock and Warm, 1989; Hancock and Ganey, 2003), recent work has shown that a performance psychology intervention which encourages conservatoire musicians' self-awareness of idiographic, optimal levels of performance energy (physiological adaptability), coupled with strategies to redirect attention away from task-irrelevant to task-relevant musical cues (psychological adaptability), successfully improves performers' capacity to manage distressing performance anxiety and boosts performance resilience (see Osborne et al., 2014).

It is even less clear whether such CB interventions have any effect upon an external observer's ratings of a performer's levels of manifest MPA. Only one study, to our knowledge, has included an external observer's perceptions of MPA, however this was not an intervention study. Kubzansky and Stewart (1999) found that, while self-reported ratings of anxiety were not associated with performance outcomes, judges' ratings of anxiety were strongly related to performance evaluations. Those who were perceived as more anxious were rated as having a poorer performance, regardless of how anxious the performers actually felt. A positive but non-significant correlation was also observed between self-perceived MPA and the judges' ratings of MPA, indicating that the performers' own ratings of anxiety did not reliably correlate with the observers' ratings.

While CB techniques have been shown to have the strongest evidence base in the management of MPA, there have also been suggestions to call upon already-established techniques in the sports psychology domain, including performance psychology and positive psychology elements, which have become popular in recent years for treating athletes with performance anxiety (Orlick and Partington, 1988; Seligman, 2002). This is largely due to the many known overlapping features of sports and musical performances, with both requiring high levels of motor control and learning, mastery over mind and body, the necessity for implicit recall and smooth performance, and the presence of an audience, which invokes the potential for enjoyment of excellence, but also psychological pressure (Yoshie et al., 2009). Performance psychology techniques place an emphasis on mental rehearsal (visualization), goal setting, focusing on strengths, with the ultimate goal being to achieve a state of “flow” or an experience of complete immersion in an activity (Williams, 2010). Positive psychology draws upon similar techniques, with a particular focus on the utilization of existing strengths, achieving flow, positive visualization, and the close study of what occurs during an optimal performance (Csikszentmihalyi, 1990).

Informed by the success of such performance and positive psychology techniques in athletes, as well as by the growing evidence base for the effectiveness of CB techniques in adult musicians, *Unleash Your Potential: Thinking Skills for Peak Performance* (Brandon and Ivens, 2009) was developed as a preventative skills-based program for adolescents to facilitate optimal performance across a wide range of performance areas. The CB elements of this program include psychoeducation, cognitive restructuring, and relaxation techniques, whilst techniques such as identification of strengths, goal-setting, positive self-talk and thinking, imagery and visualization are drawn from the performance and positive psychology literature.

Given the mounting evidence that MPA is a topic of concern for young musicians and the lack of MPA interventions for this age group, there is a need to scientifically evaluate early intervention and preventative approaches that can be delivered in the school system. The aims of this study were three-fold: (1) to assess whether Unleash Your Potential can be effective in reducing MPA in young musicians; (2) to explore whether participation in this program changes external ratings of manifest MPA; and (3) to assess whether the program improves the quality of a music student's performance. It was hypothesized that:

1. Unleash Your Potential would be superior to a wait-list (WL) control condition in reducing self-reported MPA, and that effects would be maintained at 2 month follow-up.
2. Unleash Your Potential would be superior to a WL control condition in reducing judge-rated MPA, and that these effects would be maintained at 2 month follow-up.
3. Unleash Your Potential would be superior to a WL control condition in improving performance quality, and that these effects would be maintained at 2 month follow-up.

## Materials and methods

### Participants

Sixty-two female instrumental students in Grade 7–9 (*M* = 13.78 years, *SD* = 0.85 years), from a Catholic girl's school in Melbourne, Australia volunteered to take part in this study. Instruments included piano (25%), woodwind (22%), string (16%), brass (10%), voice (10%), guitar/bass guitar (6%), and percussion (5%), with two girls combining voice with guitar (3%). Students had learned their instrument for an average of 4 years (*SD* = 2.6, range 1–10 years).

### Measures

#### Demographics

Data included age, main instrument, and years of learning the instrument. Eight open-ended questions asked participants about their reasons for joining the program, expected gains from participating and previous techniques used to manage MPA. Responses helped guide the program facilitators to meet the particular needs of students within each group.

#### Self-reported MPA

Self-reported MPA was measured using a modified version of the Music Performance Anxiety Inventory for Adolescents (MPAI-A; Osborne and Kenny, 2005; see Supplementary Material Table 1). Twelve items from the Somatic, Cognitive and Performance Evaluation factors were used to create a state-performance version. Items were modified to describe immediate feelings related to the pre-performance. For example, “Before I perform I get butterflies in my stomach” was changed to “I have butterflies in my stomach,” and “When I perform in front of an audience I get sweaty hands” was changed to “I have sweaty hands.” Items were answered using a 7-point Likert scale ranging from 0 = “Not at all; Hardly ever” to 6 = “All or most of the time” and summed to yield a total score (range = 0–72), where higher scores indicate higher levels of MPA. Similar to the original scale, this modified measure displayed high internal consistency (Cronbach's alpha = 0.91).

#### Judge-rated MPA and performance quality

Two judge-rated scales measured the behavioral manifestation of MPA and performance quality (adapted from Halls, 2011; see Supplementary Material Table 2). Behavioral manifestations of MPA were scored using a Likert scale of “0—No effect” to “6—Significant effect” where higher scores indicate higher levels of performance anxiety. For items such as sweating, trembling, hyperventilating, tense musculature, and technical mishaps/stumbling, an overall score of how much these behavioral manifestations were affecting performance was also collected. Performance quality (PQ) was graded according to a Likert scale of “0—Very poor (<45%)” to “6—Outstanding (95%+),” where higher scores indicate better performance quality. Similarly, an overall score for PQ was collected based items such as technique, dynamic contrasts, tempo/rhythm, phrasing, sound/tone, and emotional impact. These adapted measures showed moderate to high internal consistency: Cronbach's alpha MPA = 0.66, PQ = 0.86. Correlations between the two judges were treated as separate dependent variables due to low and inconsistent correlations between ratings. The correlations between judges were as follows for MPA: Time 1 *r* = 0.19 (*p* > 0.05), Time 2 *r* = 0.31 (*p* < 0.05), Time 3 *r* = 0.24 (*p* > 0.05); for PQ: Time 1 *r* = 0.69 (*p* < 0.05), Time 2 *r* = 0.66 (*p* < 0.01), Time 3 *r* = 0.41 (*p* < 0.05).

### Procedure

The Human Research Ethics Committee of the University of Melbourne approved the study. After participants completed consent forms, they were allocated to the intervention (*n* = 30) or the wait-list control group (*n* = 32) using a pseudo-randomization method. Students were listed in alphabetical order and alternately assigned to either the intervention or the wait-list control group. Two students were non-randomized due to extra-curricular commitment clashes. All participants were sent an email detailing the upcoming performance and the requirements of the piece to be performed. As outlined in the email, the piece had to be approximately 2 min long, reflect their current level of playing, and not have been previously performed in a formal setting. Both groups also completed a self-report baseline measure (approximately 1 week prior to the first performance), which assessed MPA, motivation and resilience in music learning and performance (see Osborne, 2013 for details).

After approximately 3 weeks of learning the piece, participants took part in Performance 1 whereby two external judges, blinded to the group conditions, rated the participants' behavioral manifestations of MPA and their PQ. These external judges met a minimum requirement of Master's level training on their main performance instrument, and had at least 20 years experience as music educators and adjudicators. Immediately before the performance students completed the MPAI-A (Performance) questionnaire, reflecting the finding that this is the period when musicians' apprehensions are reported to be greatest (Salmon, 1990). All performances were audio-video recorded.

Following this, the 8-week Unleash Your Music Potential program (Table 1) was conducted for the intervention group, administered in a group format by two school psychologists who are also the authors of the program. The delivery of the program was adapted from the 10 sessions outlined in the published workbook (Brandon and Ivens, 2009) to fit within the school term and study objectives. Sessions relating to team harmony and booster shots were not included for this reason. Team harmony was not included because the main outcome measure was a solo performance.

**Table 1:** **Unleash Your Music Potential session schedule**.

**Session**	**Title**	**Themes**
1	Discover the champion within	Peak performance, being the boss of your own thinking, personal strengths
2	Create your future	Goals, goal setting, motivation
3	Success is a mindset	Success, self-talk, feelings, affirmations
4	It's all in the preparation	Routines, relaxation, self-talk, stress reduction
5	The ultimate dress rehearsal	Mental imagery, mental rehearsal, visualization
6	Peak condition	Stress management, wellbeing
7	The achievement zone	Focus, flow, performing “in the zone”
8	Back on track and energized	Setbacks, loss, disappointment, resilience, coping, positive thinking

**Figure 1:**
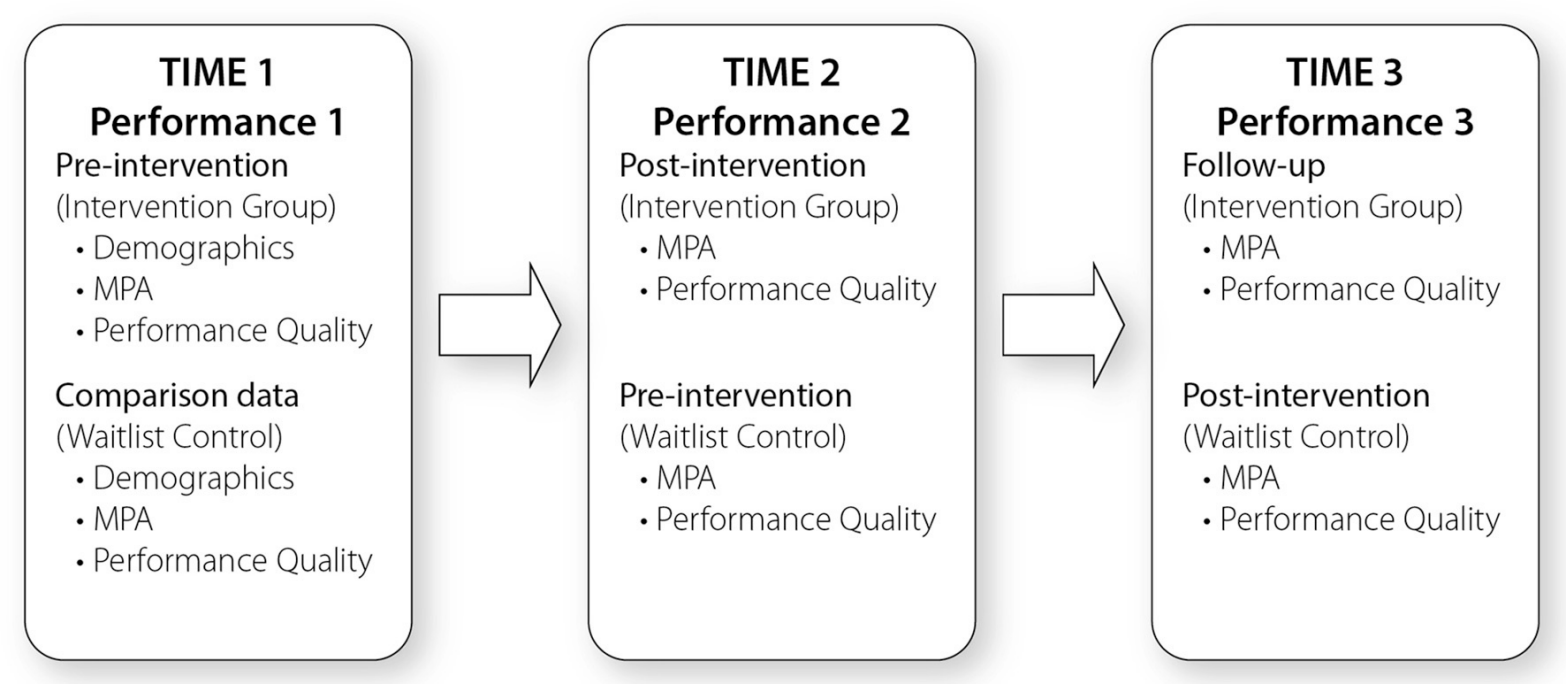
**Study design**.

After this 8-week period students in both groups participated in Performance 2, using the same procedure as Performance 1. The wait-list control group then took part in a repeated delivery of the 8-week Unleash Your Music Potential program during the subsequent school term. Performance 3 took place following this intervention, serving as the post-intervention measure for the wait-list control as well as a 2-month follow-up measure for the intervention group. The overall time span of the study from Time 1 to Time 3 was 5.5 months (see Figure 1).

### Data analysis

Dependent variables were checked for assumptions of normality using the Kolmogorov-Smirnov (K-S) statistic. Assumptions for normality were met for self-rated MPA for both groups at time 1 and 2, and the waitlist control group at time 3. Self-reported MPA of the intervention group violated assumptions at time 3 (K-S = 0.03). This data was included in parametric analyses, as Levene's and Mauchly's tests were not violated. Mixed-model 2 (group) × 3 (time) ANOVAs were conducted to assess the effect of the intervention on decreased MPA (self-rated and judge-rated) and improved PQ. Difference between groups at time 2 were analyzed using independent group *t*-tests. Maintenance of effects at 2 month follow-up (time 3) were assessed using repeated measure *t*-tests. Between and within-group effect sizes were measured by eta-squared.

Judges' ratings were not highly correlated and are reported separately in the results. Assessment of judge-rated MPA and PQ at each of the three performance times revealed violations of normality for both judges' ratings of MPA and PQ at each performance. When scores were converted to Z scores, this resulted in only three variables being violated (Judge 2-rated MPA at time 1 and 3, Judge 1-rated MPA at time 2). Further investigation of these three variables using Levene's test of Homogeneity of variance revealed that the variances were equal between groups in these variables, respectively [*F*_(1, 60)_ = 1.04, *ns*; *F*_(1, 60)_ = 0.001, *ns*; *F*_(1, 60)_ = 2.29, *ns*]. Therefore, ANOVAs were conducted for each judge separately on judge-rated MPA and PQ.

Students who failed to attend two out of three performances were excluded from analyses (*n* = 4). Data for students who missed one performance (*n* = 13) were substituted using the mean of their group (intervention or wait-list control) as analyses revealed no significant difference between cases present and cases absent on age, years of playing, and the baseline self-report measure of MPA.

## Results

### Descriptive statistics

Baseline characteristics of each group, including age, years of learning instrument, self-rated MPA and judge ratings of MPA and performance quality indicate that the two groups were comparable, as shown in Table 2. Baseline levels of MPA as measured by the full 15-item MPAI-A measure were within the mean range reported by three studies investigating MPA in females in this age group: range *M* = 38.24–56.45; *SD* = 15.21–20 (Osborne and Kenny, 2005; Osborne et al., 2005; Thomas and Nettelbeck, 2014).

**Table 2:** **Baseline characteristics by group**.

	**Intervention [***n*** = **30**, *M* (*SD*)]**	**Wait-list control [***n*** = **32**, *M* (*SD*)]**	***p*-value**
Age	13.70 (0.87)	13.87 (0.84)	0.45
Years playing	3.96 (2.77)	4.10 (2.40)	0.85
MPAI-State self-rated	28.69 (10.12)	26.69 (10.60)	0.45
MPAI-A full scale	44.86 (13.61)	42.37 (15.14)	0.52
**MPA JUDGE-RATED**			
Judge 1	1.80 (0.10)	1.79 (0.82)	0.98
Judge 2	0.38 (0.55)	0.31 (.051)	0.61
**PERFORMANCE QUALITY**			
Judge 1	2.43 (1.01)	2.60 (0.90)	0.49
Judge 2	3.20 (1.32)	3.60 (1.03)	0.19

MPA, Music performance anxiety. The MPAI-A full-scale score is provided to assist comparisons of the characteristics of this sample to other studies using the full-scale. The effect of the intervention on the full-scale MPAI-A are discussed in Osborne (2013).

### Hypothesis 1: unleash your potential reduces self-reported MPA

Preliminary analyses revealed that there were no significant differences between the intervention and wait-list control groups' self-reported MPA levels at time 1, *t*_(60)_ = 0.76, *p* > 0.05, or time 3, *t*_(60)_ = −1.22, *p* > 0.05. There were also no significant differences within the intervention group across time 2 and 3, *t*_(29)_ = 1.6, *p* > 0.05, and within the wait-list control group across time 1 and 2, *t*_(31)_ = 0.63, *p* > 0.05. Therefore, initial analyses investigated the overall effect of the intervention by collapsing pre- or post-treatment values for the wait-list control and intervention group respectively. This showed that the intervention was successful in significantly reducing self-reported MPA [pre-treatment *M* = 27.39, *SD* = 9.80, post-treatment *M* = 18.90, *SD* = 8.44, *F*_(1)_ = 45.51, *p* < 0.001, η^2^ = 42.7%].

**Figure 2:**
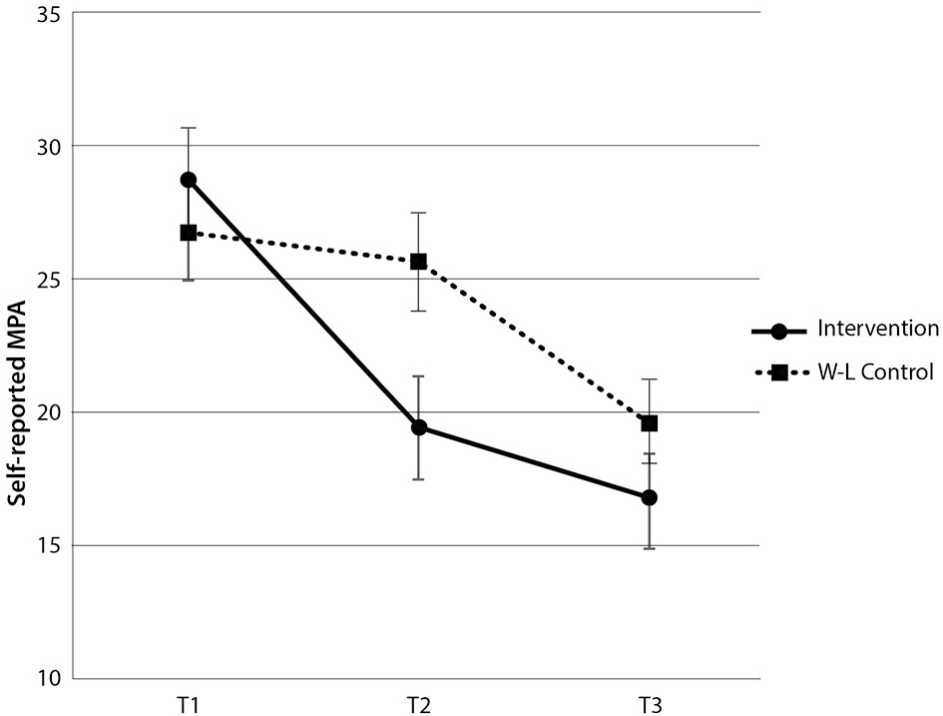
**Self-rated MPA scores by group and time**. Note these are mean scores with standard error bars, where higher scores indicate more anxiety.

The mixed model ANOVA revealed a significant interaction between group and time [*F*_(2, 120)_ = 4.99, *p* < 0.01, η^2^_*p*_ = 7.7%]. Assessment of mean values, shown in Figure 2, revealed that the intervention group gained significant benefit from the intervention between time 1 and time 2 (pre vs. post), and the wait-list control group showed a significant benefit from the intervention between time 2 and time 3 (pre vs. post). This effect was supported by the main effect of time [*F*_(2, 120)_ = 26.35, *p* < 0.001, η^2^_*p*_ = 30.5%] (see Figure 2). There was no main effect for group [*F*_(2, 120)_ = 1.37, *p* > 0.05]. *Post-hoc* analyses showed a significant reduction in self-reported MPA for the intervention group between time 1 and time 2 [*t*_(29)_ = 4.34, *p* < 0.001], and for the wait-list control group, between time 2 and time 3 [*t*_(31)_ = 3.67, *p* < 0.01]. The intervention group (post-intervention) showed significantly less self-reported MPA than the wait-list control group (pre-intervention) at time 2 [*t*_(60)_ = −2.4, *p* < 0.05].

Sustained benefits of the program were demonstrated by the significant reduction in MPA for the intervention group between time 1 and 3 [*t*_(29)_ = 5.35, *p* < 0.001] and a maintenance of this reduced level of MPA from time 2 to time 3 [*t*_(29)_ = 1.60, *p* > 0.05]. Overall, this indicates that the intervention was highly effective in reducing self-reported MPA in both groups and that the reduced levels of MPA were maintained 2 months after students completed the program. These results demonstrate that the intervention led to a significant reduction in self-reported MPA, which was sustained for 2 months post-completion of the intervention.

### Hypothesis 2: unleash your potential reduces judge-rated MPA

**Figure 3:**
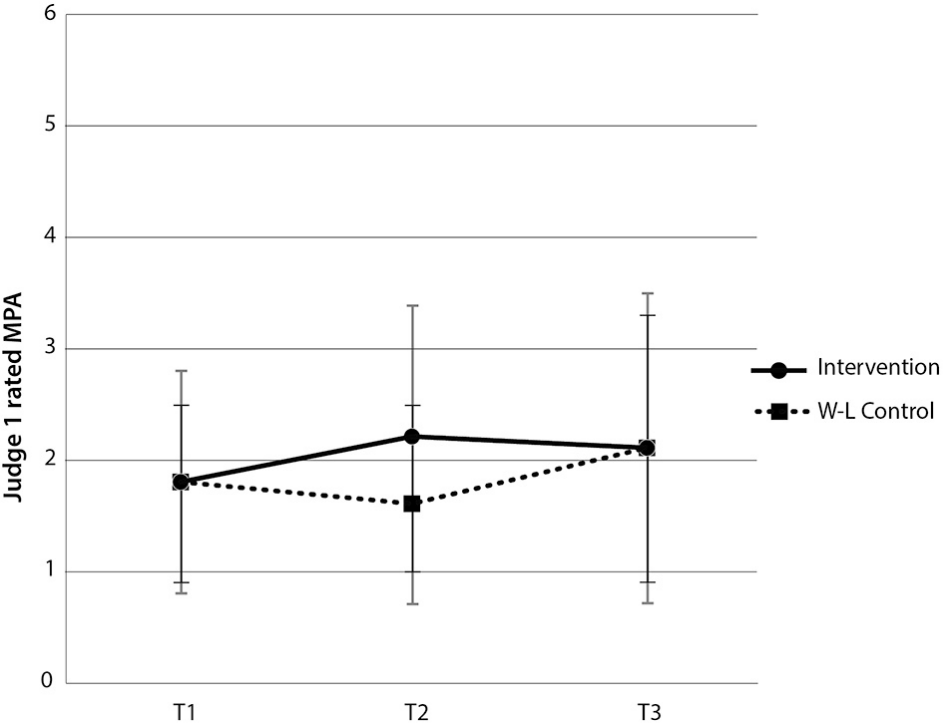
**Judge 1-rated MPA scores by group and time**. Note these are mean scores with standard error bars, where higher scores indicate more anxiety.

The intervention had no therapeutic benefit in terms of Judge 1's ratings of behavioral manifestations of MPA, as the interaction between group and time [*F*_(2, 120)_ = 2.64, *p* > 0.05], and main effects of time [*F*_(2, 120)_ = 1.54, *p* > 0.05] and group [*F*_(1, 60)_ = 1.15, *p* > 0.05] were all non-significant (see Figure 3).

**Figure 4:**
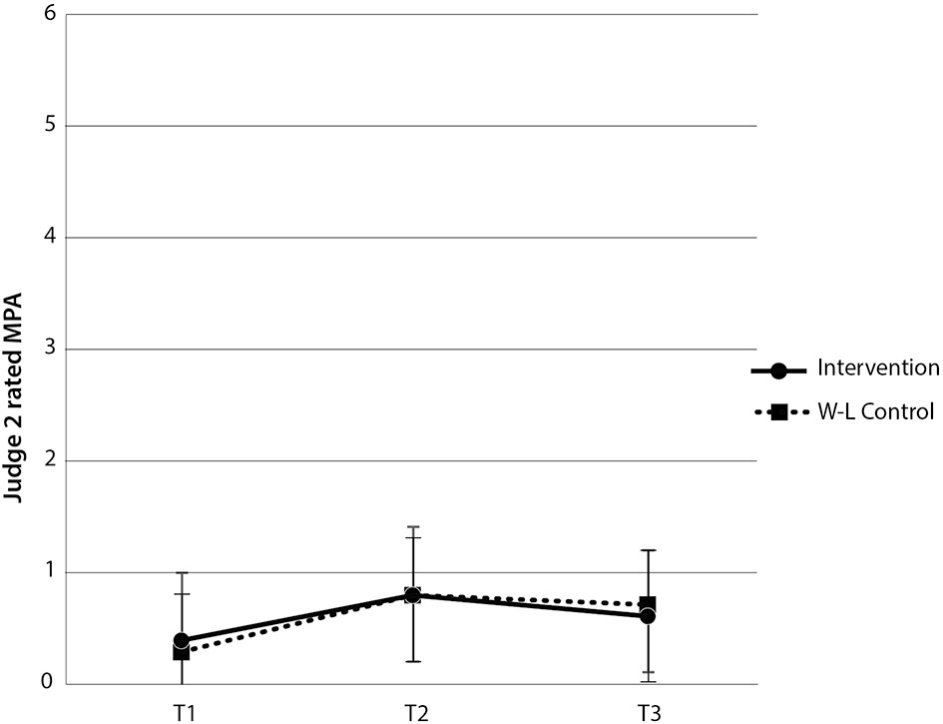
**Judge 2-rated MPA scores by group and time**. Note these are mean scores with standard error bars, where higher scores indicate more anxiety.

For Judge 2's MPA ratings, the interaction between group and time [*F*_(2, 120)_ = 0.40, *p* > 0.05] and main effect for group [*F*_(1, 60)_ = 0.001, *p* > 0.05] were both non-significant. There was, however, a main effect of time [*F*_(2, 120)_ = 12.73, *p* < 0.001, η^2^_*p*_ = 17.51%]. Paired-samples *t*-tests revealed a significant increase in Judge 2's rating of MPA for the intervention group between time 1 and time 2 [*t*_(29)_ = −3.28, *p* < 0.05], and no significant difference in Judge 2's ratings of MPA for the wait-list control group, between time 2 and time 3 [*t*_(31)_ = 1.25, *p* > 0.05] (see Figure 4). There was no main effect for group [*F*_(1, 60)_ = 0.001, *p* > 0.05]. While paired-samples *t*-tests indicated that there was a statistically significant reduction in Judge 2's ratings of MPA for the intervention group between time 2 and 3, these ratings were higher than the baseline rating, indicating that the intervention had no therapeutic benefit in terms of Judge 2's ratings of behavioral manifestations of MPA.

Contrary to expectation, the intervention group demonstrated significantly more MPA than the wait-list control at time 2 [*t*_(60)_ = 2.47, *p* < 0.05]. In addition, Judge 2 did not find any significant difference in MPA between groups at time 2 [*t*_(60)_ = 0.20, *p* > 0.05]. Therefore, levels of behaviorally manifested MPA were equivalent across both groups despite one group having undertaken the intervention. The combined within and between-groups results demonstrate that the intervention did not reliably lead to reductions in judge-rated behavioral manifestations of MPA in music students, both immediately following the intervention and at 2 month follow-up. Notably, however, given these judge ratings of MPA were consistently low, a floor effect might exist, precluding meaningful reductions in judge-rated MPA.

### Hypothesis 3: unleash your potential increases judge-rated performance quality

**Figure 5:**
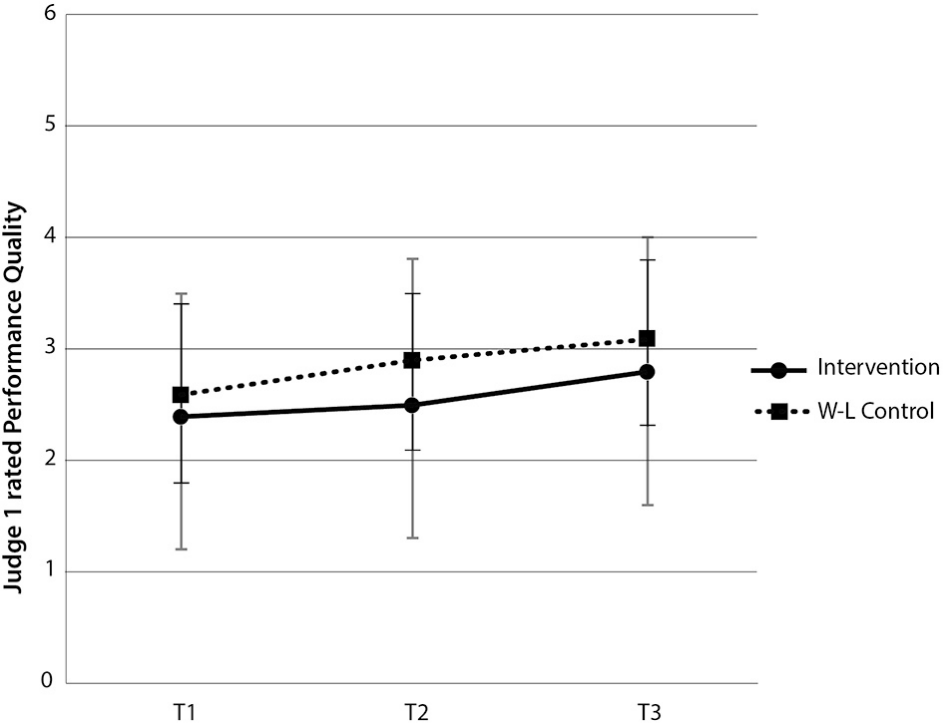
**Judge 1-rated performance quality scores by group and time**. Note these are mean scores with standard error bars, where higher scores indicate better performance quality.

For Judge 1's PQ ratings, there was no significant interaction between group and time [*F*_(2, 120)_ = 0.48, *p* > 0.05] nor main effect for group [*F*_(1, 60)_ = 2.63, *p* > 0.05]. There was, however, a main effect of time [*F*_(1, 120)_ = 7.08, *p* < 0.05, η^2^_*p*_ = 10.6% (see Figure 5)]. Paired-samples *t*-tests revealed no significant difference in Judge 1's rating of PQ for the intervention group between time 1 and time 2 [*t*_(29)_ = −0.403, *p* > 0.05], and no significant difference in Judge 1's ratings of PQ for the wait-list control group, between time 2 and time 3 [*t*_(31)_ = −1.67, *p* > 0.05]. There was a statistically significant increase in Judge 1's ratings of PQ for the intervention group between time 1 and 3 [*t*_(29)_ = −2.53, *p* < 0.05] (see Figure 5), indicating that, despite the intervention having had no immediate significant therapeutic benefit in terms of Judge 1's ratings of PQ, there was a significant increase in PQ over the five and half month period of the study.

**Figure 6:**
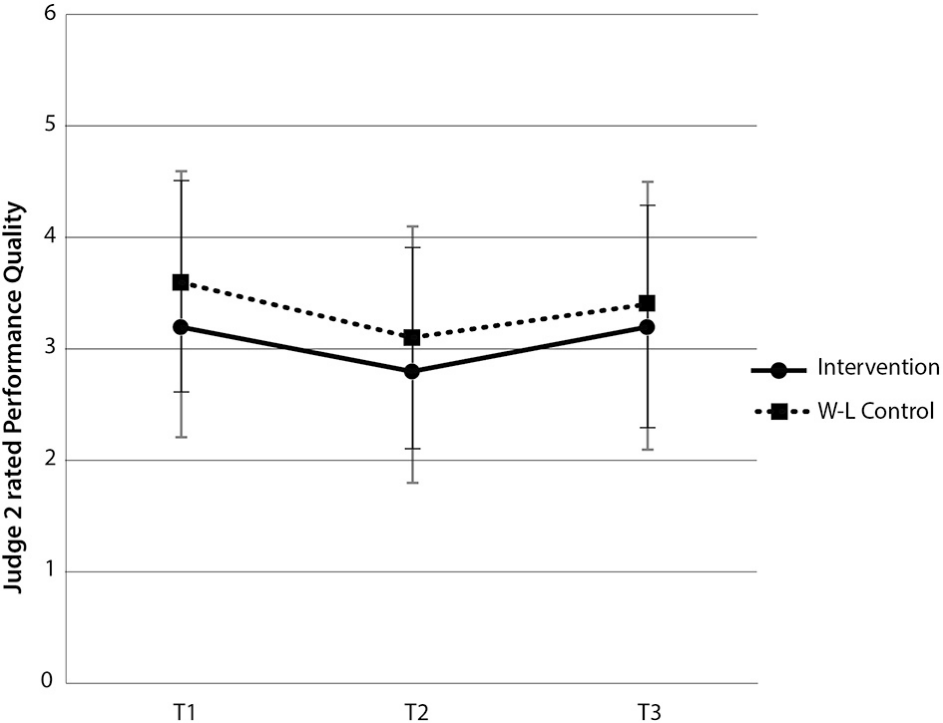
**Judge 2-rated performance quality scores by group and time**. Note these are mean scores with standard error bars, where higher scores indicate better performance quality.

For Judge 2's performance quality ratings, both the interaction between group and time [*F*_(2, 120)_ = 0.36, *p* > 0.05] and main effect for group were not significant [*F*_(1, 60)_ = 1.36, *p* > 0.05]. There was however a main effect of time [*F*_(2, 120)_ = 6.10, *p* < 0.05, η^2^_*p*_ = 9.2%]. There was no support for improved ratings of PQ for the intervention group between time 1 and time 2 [*t*_(29)_ = 1.62, *p* > 0.05], yet there was a significant increase in Judge 2's ratings of PQ for the wait-list control group between time 2 and time 3 [*t*_(31)_ = −2.04, *p* < 0.05]. While a paired-samples *t*-test indicated a statistically significant increase in Judge 2's ratings of PQ for the intervention group between time 2 and 3 [*t*_(29)_ = −2.09, *p* < 0.05], ratings of PQ at time 1 and 3 were equivalent [*t*_(29)_ = −0.21, *p* > 0.05] (see Figure 6). These findings indicate that Judge 2 observed an immediate beneficial effect of the intervention on PQ for the wait-list control group and a delayed improvement in PQ from time 2 for the intervention group.

At time 2, both judges rated the level of PQ as equivalent, despite one group having undertaken the intervention [Judge 1: *t*_(60)_ = −1.68, *p* > 0.05; Judge 2 *t*_(60)_ = −0.86, *p* > 0.05]. These combined within and between-groups results for hypothesis 3 provide partial support for an improvement in PQ immediately following the intervention and across the full five and a half month research period.

## Discussion

This study provides preliminary evidence that an 8-week psychological skills program which teaches the cognitive skills essential for optimal performance in a group format is effective in reducing self-rated MPA in adolescent musicians and is superior to a waitlist control condition. Compared to baseline, participants in both groups demonstrated significantly less self-reported MPA when they had completed the intervention. Moreover, this reduction in MPA was sustained 2 months after the intervention had been completed, indicating its potential for longer-term therapeutic benefits. Furthermore, the intervention group reported significantly less MPA than the wait-list control group at time 2 (intervention group: post-intervention and wait-list control group: pre intervention), adding further weight to evidence that this intervention has a beneficial effect upon students' self-perceived levels of MPA. These findings were accompanied by large effect sizes. This result is consistent with the research of Osborne et al. (2007), who found a CB program incorporating performance psychology techniques to be effective in reducing adolescent student's self-reports of MPA. Apart from Osborne (2013), to our knowledge no other studies have examined the efficacy of a CB intervention for adolescent musicians, although there have been numerous studies that have found such interventions to be effective in reducing MPA in adult musicians (Harris, 1987; Clark and Agras, 1991; Roland, 1993; Connolly and Williamon, 2004; Buswell, 2006). It is not surprising then that CB interventions also show promise in helping reduce MPA in adolescents given the qualitatively similar experience of MPA between adults and adolescents.

The intervention, however, did not reliably lead to a decrease in judge ratings of behavioral manifestations of MPA in students. This was true both immediately following the intervention and at 2-month follow-up. This finding should be interpreted with caution given the consistently low ratings of MPA by both judges. Overall, the judges did not appear to detect behavioral signs of MPA in the students, regardless of whether the students had undertaken the intervention or not. This finding conveys an important psychoeducational message: while students may feel anxious and/or perceive themselves to look anxious, this anxiety is not necessarily discernable to audience members, even when these members of the audience have been explicitly requested to be vigilant to signs of MPA. This finding is consistent with a study by Kubzansky and Stewart (1999) in which performer-rated state anxiety and judge-rated MPA during an orchestral audition process were not significantly related.

A second psychoeducational message drawn from the poor inter-rater reliability in the judges' ratings of MPA is that one observer's perception of a performer's anxiety can be very different from another's, and therefore is subjective and not necessarily under the performer's control. Thus, while the intervention was not found to reliably reduce judge-rated MPA, these findings highlight the potential discrepancy between how much a performer vs. an observer perceives MPA to be present and, ultimately, point to the subjective nature of observer-rated MPA.

In addition, this study found inconsistent support for improved performance quality as a result of participating in the intervention. These findings highlight the subjective and variable nature of an observer's judgment of another's performance. A wide discrepancy between judges' ratings has also been found in other studies employing judge-rated performance quality. For example, Reitman (2001) found such wide inter-rater discrepancies that it was impractical to attempt further analysis on the performance quality variable, measured using error count. Similarly, Clark and Williamon (2011) found such low correlations between judge-rated performance quality on eight items pertaining to overall quality, technical proficiency, musical understanding, communicative ability, level of preparedness, self-assuredness, interpretative imagination and originality, and ability to cope with performance stress, that they deemed this measure invalid and excluded it from their main analyses.

Our finding is also consistent with previous studies that failed to find significant improvements in performance quality following CB interventions when targeting MPA (Wardle, 1975; Mansberger, 1988; Deen, 2000; Osborne et al., 2007). One possible explanation for this finding is that CB interventions, like Unleash Your Potential, have been designed to target subjective symptoms of MPA (cognitive, behavioral and physiological), rather than to optimize performance quality. Certainly, the aim of the present study's intervention was to make performance less psychologically distressing and ultimately more enjoyable for the individual, rather than to achieve excellence in performance quality *per se*. A second explanation, as suggested by Reitman (2001) who reported a similar finding, could be that the relatively short period of time that students had to translate newly learnt coping skills into the performance setting meant that behavioral changes were yet to be demonstrated. This is consistent with other studies that found behavioral changes to lag behind cognitive changes post-treatment (Appel, 1976). Thus, while the intervention did not reliably lead to improvement in students' performance quality, this finding highlights the specificity of the intervention in targeting self-perceived MPA, and in rendering students' experiences of performing more enjoyable, over and above performance quality.

While this study demonstrated that a CB intervention program with performance and positive psychology elements shows great promise in helping adolescent music students reduce their experience of feeling anxious during performance, it was beyond the scope of this study to examine specific elements of the program that could account for this therapeutic benefit. It may be that a combination of cognitive, behavioral, performance and positive psychological strategies effected this change in MPA levels. Future studies should attempt to parse out the particular mechanisms that drive improvements in self-rated MPA and compare the impact of each on the cognitive, physiological and behavioral manifestations of MPA.

This study also illuminated some of the inherent difficulties in measuring externally-rated behaviors like MPA and performance quality. The lack of inter-rater reliability on both of these variables points to the challenge of reliably measuring (1) physiological signs of MPA that are subtle and thus tend to be apparent to the performer only, and (2) an outcome variable like performance quality which can be notoriously subjective. Given that the judges in the present study were sitting at least 5 m away from the performers, future studies should endeavor to take “close up” video footage of each of the performers, which could potentially make it easier for observers to detect MPA manifestations like trembling, sweating, and blushing. While it is a difficult task to overcome the problem of variability between observers' ratings of performance quality, future researchers should endeavor to train judges to an adequate level of inter-rater reliability.

It is also possible that the performance task used in our study had limited ecological validity due to the fact that this particular performance did not contribute to school assessments. Future studies should endeavor to incorporate performances that are delivered as part of the school assessment program, in order to potentially evoke higher levels of MPA. Finally, given the mixed findings in the literature regarding the efficacy of CB interventions for MPA to facilitate improvements in performance quality, future investigators should continue to look at the relationship between reduced MPA and performance quality. They should examine whether longer-term interventions might allow for behavioral changes in performance quality to be demonstrated, once participants have had a greater period of time to assimilate coping skills.

We have demonstrated that a CB intervention program can lead to significant reductions in self-perceived performance anxiety in adolescent music students. We also highlight an important and reassuring psychoeducational message—that while you may feel anxious during a performance, it is very unlikely that the audience can detect this. Given the growing evidence for the ubiquity of MPA in children and adolescents, the young age at which the majority of musicians begin their training, and the potential career-devastating effects of untreated MPA in older musicians, it is critical that evidence-based intervention programs, like *Unleash Your Potential*, are incorporated into school music curricula. This could result in a more well-rounded approach to music education, whereby the goal of musical excellence is balanced by an equal emphasis upon self-perceived confidence, and ultimately, enjoyment in music performance.

### Conflict of interest statement

The authors declare that the research was conducted in the absence of any commercial or financial relationships that could be construed as a potential conflict of interest.
