# Supplementary material for: Psychological intervention reduces self-reported performance anxiety in high school music students
Source: Front Psychol. 2015 Mar 3;6:195. doi: 10.3389/fpsyg.2015.00195 (PMC4347450; doi:10.3389/fpsyg.2015.00195)
Supplement: Supplementary file 1 [file Table1.PDF]

Music Performance Anxiety Inventory for Adolescents (State, Performance)

(MPAI-A-State; Osborne, 2012)

Published in Braden AM, Osborne MS and Wilson SJ (2015). Psychological intervention reduces self-reported performance anxiety in high school music students. *Front. Psychol.* **6**:195. doi: 10.3389/fpsyg.2015.00195

WHAT I THINK ABOUT PERFORMING MUSIC

A number of statements which people use to describe themselves before a performance are given below.

Please read each statement and then select the number that indicates how you feel RIGHT NOW, AT THIS VERY MOMENT.

There are no right or wrong answers. Do not spend too much time on any one statement but give the answer which seems to describe your present feelings best.

		Not at all						All of the time
1.	I have butterflies in my stomach.	0	1	2	3	4	5	6
2.	I am worried about my ability to perform.	0	1	2	3	4	5	6
3.	I am trembling and/or shaking.	0	1	2	3	4	5	6
4.	I am afraid of making mistakes.	0	1	2	3	4	5	6
5.	My heart is beating very fast.	0	1	2	3	4	5	6
6.	I am finding it hard to concentrate on my music.	0	1	2	3	4	5	6
7.	If I make a mistake during this performance, I will panic.	0	1	2	3	4	5	6
8.	I have sweaty hands.	0	1	2	3	4	5	6
9.	I feel nervous.	0	1	2	3	4	5	6
10.	I am worried that the judges will not like my performance.	0	1	2	3	4	5	6
11.	My muscles feel tense.	0	1	2	3	4	5	6
12.	I think I will be happy with this performance when I am finished.	6	5	4	3	2	1	0

Music Performance Anxiety Inventory for Adolescents (Trait)

(MPAI-A; Osborne & Kenny, 2005)

Published in Osborne, M. S., & Kenny, D. T. (2005). Development and validation of a music performance anxiety inventory for gifted adolescent musicians. *Journal of Anxiety Disorders*, *19*(7), 725-751. doi: 10.1016/j.janxdis.2004.09.002

WHAT I THINK ABOUT PERFORMING MUSIC

A number of statements which people use to describe how they feel when they perform are given below.

Please think about music in general and your major instrument and answer the questions by circling the number which describes how you GENERALLY feel.

There are no right or wrong answers. Do not spend too much time on any one statement but give the answer which seems to describe your feelings best.

		Not at all						All the time
1.	Before I perform I get butterflies in my stomach.	0	1	2	3	4	5	6
2.	I often worry about my ability to perform.	0	1	2	3	4	5	6
3.	I would rather play on my own, than in front of other people.	0	1	2	3	4	5	6
4.	Before I perform, I tremble or shake.	0	1	2	3	4	5	6
5.	When I perform in front of an audience, I am afraid of making mistakes.	0	1	2	3	4	5	6
6.	When I perform in front of an audience, my heart beats very fast	0	1	2	3	4	5	6
7.	When I perform in front of an audience, I find it hard to concentrate on my music.	0	1	2	3	4	5	6
8.	If I make a mistake during a performance, I usually panic.	0	1	2	3	4	5	6
9.	When I perform in front of an audience I get sweaty hands.	0	1	2	3	4	5	6
10.	When I finish performing, I usually feel happy with my performance.	6	5	4	3	2	1	0
11.	I try to avoid playing on my own at a school concert.	0	1	2	3	4	5	6
12.	Just before I perform, I feel nervous.	0	1	2	3	4	5	6
13.	I worry that my parents or teacher might not like my performance.	0	1	2	3	4	5	6
14.	I would rather play in a group or ensemble, than on my own.	0	1	2	3	4	5	6
15.	My muscles feel tense when I perform.	0	1	2	3	4	5	6
