# Supplementary material for: Psychological intervention reduces self-reported performance anxiety in high school music students
Source: Front Psychol. 2015 Mar 3;6:195. doi: 10.3389/fpsyg.2015.00195 (PMC4347450; doi:10.3389/fpsyg.2015.00195)
Supplement: Supplementary file 2 [file Table2.PDF]

Published in Braden, A., Osborne, M. S., & Wilson, S. J. (2015). Psychological intervention reduces self-reported performance anxiety in high school music students. *Frontiers in Psychology (Cognitive Science)*.

Expert Judge Ratings

Judge: ______________________________ Date: ___________

Student name: ______________________________

Instrument: ______________________________

Piece: ______________________________

Performance Quality

Rank each area of performance from 0 (very poor) to 6 (outstanding)

	Very poor						Out-standing
Technique (includes fingering, articulation, bowing, breathing, tone production)	0	1	2	3	4	5	6
Dynamic Contrasts	0	1	2	3	4	5	6
Tempo/Rhythm	0	1	2	3	4	5	6
Phrasing	0	1	2	3	4	5	6
Sound/Tone	0	1	2	3	4	5	6
Emotional impact	0	1	2	3	4	5	6
Overall Grade	0	1	2	3	4	5	6
	<45%	46-54%	55-64%	65-74%	75-84%	85-94%	95%+

Behavioural manifestation of MPA

Rank each area of the performance from 0 (not obvious) to 6 (extremely obvious)

	Not obvious						Extremely obvious
Sweating	0	1	2	3	4	5	6
Trembling	0	1	2	3	4	5	6
Breathing (hyperventilating)	0	1	2	3	4	5	6
Tense musculature	0	1	2	3	4	5	6
Technical mishaps/stumbling	0	1	2	3	4	5	6
	No effect						Significant effect
How much are the above affecting performance?	0	1	2	3	4	5	6

Any other comments on the performance?

____

____

____
